# Digital Learning, Life Satisfaction, and Perceived Stress Due to COVID-19 Emergency: Case Study Among Female Saudi University Students

**DOI:** 10.3389/fpsyg.2022.875608

**Published:** 2022-05-31

**Authors:** Fatma Mabrouk, Mohamed Mehdi Mekni, Aishah Aldawish

**Affiliations:** ^1^Department of Economics, College of Business and Administration, Princess Nourah bint Abdulrahman University, Riyadh, Saudi Arabia; ^2^Faculty of Law, Economics and Management Sciences of Jendouba, University of Jendouba, Jendouba, Tunisia

**Keywords:** digital learning, life satisfaction, stress, defense mechanisms, coping strategies, COVID-19

## Abstract

The paper explores the impact of the corona virus disease-19 (COVID-19) pandemic on the Saudi higher education system. The research focuses on the relationship between digital learning in COVID-19 time, life satisfaction, and stress among female students. The study discusses measures, practices, defense mechanisms, and coping strategies to face challenges. Using an online survey based on psychological effects and its role in defense mechanisms and coping strategies, findings show that digital learning provides flexibility in terms of time and offers resources at a lower cost compared to traditional learning. In addition, results show that the coping strategy perception is higher in obtaining a good score and succeeding than to get over the pandemic and recovering from the illness itself. Finally, results confirm that a positive attitude influences positively life satisfaction.

## Introduction

The corona virus disease-19 (COVID-19) pandemic hits the Word by surprise and impacts all the countries as well as sectors. Therefore, an acceleration of the digital transformation of many sectors and especially higher education are quickly happening. Consequently, there has been an increased interest in how managing mental health in the way that the pandemic is transforming the lives of people as well as the university life of the students. Some studies on the mental health consequences of living through the COVID-19 pandemic are conducted (Aknin et al., 2021). Different foundations and institutions have addressed how to manage anxiety, including helpful wellness tips. For example, the establishment of counseling mechanisms that leaves you keeping control, doing sports, and breathing exercises, other than the availability of people who make you feel safe and loved.

The Saudi Vision 2030 and the National Transformation Program implement an ambitious roadmap for education transformation in the Kingdom of Saudi Arabia. According to Saudi Arabia’s 2022 budget allocation, education remains to receive the principal portion of the government budget spending around 20% of the budget, i.e., SR185 billion out of a total budget of SR955 billion. Moreover, Saudi Arabia is the largest information and technology marketplace in the GCC Region. Overall expenditure on information and communication technology reaches $37 billion in 2020, up 2.4% in 2019 (Gori et al., 2020). Technological infrastructure development is a prerequisite to promoting the rapid and easy implementation of digital education.

Since the closure of schools and universities imposed by governments around the world to curb the COVID-19 pandemic, the use of digital tools and platforms has become the only way to ensure the continuity of teaching and learning. Thus, emergency distance learning has been imposed on teachers and learners. Many studies tried to analyze the opportunities and challenges of emergency remote teaching based on the experience of the COVID-19. They highlight different challenging aspects.

Technical problems (insufficient, inadequate computer laboratories, unreliability, and unstable Internet connections) and lack of necessary electronic devices (computers, laptops, tablets, and smart devices) are still the major barriers to digital learning. Ferri et al. (2020) find out that students and teachers have encountered different obstacles in distance learning due to existing limitations related to technology. Zalat et al. (2021) show that technological challenges, such as connectivity issues and shortage of computer equipment are the main difficulties/obstacles. All the same, Shim and Lee (2020) explore South Korean college students’ experiences. Survey results show that students noted some positive points of emergency distance education but they complained about the instability of the connection. Adnan and Anwar (2020) explore Pakistani students’ (Undergraduate and postgraduate) attitudes toward mandatory distance-learning courses. They find online learning inefficient in underdeveloped countries and particularly in Pakistan because a large majority of students cannot access the internet due to technical and financial problems.

Another problem that has been revealed is the lack of interactions with the instructor and how to promote interaction between teachers and students as well as among students (Adnan and Anwar, 2020; Shim and Lee, 2020). Ferri et al. (2020) recommend introducing innovations in teaching methods to remedy shortcomings observed. One of the challenges, as well, is digital learning financing, specifically the efficiency of government spending and private investment on how to improve digital learning funding, a clear understanding of the vision and mission of public and private educational institutions is required (Paul and Jefferson, 2019).

Unfortunately, the subject of well-being and life satisfaction challenges is not so much explored. Studies exploring stress and burnout in the occupational and educational settings were published briefly and explore stress and burnout associated with the new model of digital living in general. During the COVID-19 pandemic, the work and education places are transformed upon lockdown application. Suitable occupations moved to remote working and telecommunication. As well, education shifted to online mode and distance learning (Mheidly et al., 2020). Research conducted on the subject of well-being and how chronic workplace stress cannot be successfully managed during the COVID-19 pandemic mainly focused on teachers, nurses, and other health and social care workers (See et al., 2020; Bou-Hamad et al., 2021; De Kock et al., 2021; Duncan and Smart, 2021; Vera San Juan et al., 2021).

The current COVID-19 pandemic has spread among societies and it affects people and particularly students physically, and psychologically (stress, depression, or psychiatric pathologies). A study conducted by Chinna et al. (2021) examines the psychological impact of the COVID-19 pandemic on university students in Asia. The study looked at students from different countries, such as Saudi students. The authors applied Zung’s self-rating anxiety scale and questions on coping strategies and found that the main stressors factors are economic restraints, remote online learning, uncertainty about educational performance, and future career projections. Adaptive coping strategies are characterized by personalities and how to face difficulties by adopting problem-solving, seeking social and emotional support that helps to diminish stress, stimulate psychological well-being, and strength overall health. Chinna et al. (2021) show that female students in Saudi Arabia experienced significantly higher levels of anxiety than their male counterparts.

This article explores the case of Saudi higher education and how the different adjustments implemented to maintain the academic activities continuity impact the students’ wellbeing. The announcement of the temporary closure of universities in Saudi Arabia, made by March 2020 changed the educational paradigm. Moving suddenly to remote teaching, with limited experiences in digital learning, whether for teachers or students caused serious impacts on life satisfaction. In these circumstances, students’ life satisfaction is at risk with youth anxiety about the COVID-19 pandemic rising and letting young people feel physically isolated and sometimes confused. In order to explore COVID-19 impact on the Saudi higher education, a questionnaire was shared with Princess Nourah Bint Abdulrahman (PNU) University’s students to gather valuable information.

There will be three research questions investigated:

H1: Is there a significant difference in the effectiveness, costs, capabilities, and educational resources between digital learning and traditional learning?

H2: Is there a significant difference in defense mechanisms and coping strategies among female students in confronting COVID-19 and digital learning?

H3: Is there a significant influence of attitudes on female students’ life satisfaction during COVID-19?

The study tries to explain the relationship between life satisfaction, stress, and coping styles among female students during the COVID-19 pandemic and aims to explore more precisely the defense mechanisms and the effectiveness of educational resources to face the unexpected health crisis. This research is expected to contribute to the extant literature by enriching our understanding of the serious impacts of COVID-19 pandemic on youths’ mental health as well as providing some useful information and recommendation for policymakers to prevent the occurrence of psychological crisis among university students. It will underwrite the connection between life satisfaction, digital learning, COVID-19, and gender in Saudi higher education by using primary and original data collected during the academic year 2020–2021. The article is structured as follows: Section 2 introduces the data and the methodology and section 3 explores and discusses the results. Section 4 determines the main conclusion, limitation, and future suggestions.

## Materials and Methods

### Participants

Participants were Bachelor’s degree students at Princess Nourah Bint Abdulrahman University (PNU). PNU is a public women’s university located in Riyadh, the capital of Saudi Arabia. It is the largest women’s university in the world. We collected 200 responses, given that the required simple is 96 students based on the following hypotheses:

**Table:** 

The confidence level	95%
The margin of error	10%
Population proportion	50%
Population size^1^	22,344

*^1^PNU students from 1 to 4 years for the academic year 2020–2021.*

The majority of participants’ ages ranged from 18 to 25 (97.5% of the participants) and lived with their families (98.5% of the participants). The sample was exclusively Saudi students.

The participants are distributed among 3 main majors 67.5% are scientific, 24.5% are humanity disciplines, and 8% are healthy. 22.5% are admitted in the first year, 15% in the second year, 27% in the third year, and 35% in the fourth year (Table 1).

**TABLE 1:** Demographic characteristics.

Age	(18–25)	195	97.50%
	<18	2	1.00%
	>25	3	1.50%
Major	Scientific	135	67.50%
	Healthy	16	8.00%
	Humanity	49	24.50%
Educational level	First year	45	22.50%
	Second year	30	15.00%
	Third year	54	27.00%
	Fourth year	71	35.50%
Living status	With family	197	98.50%
	University compound	3	1.50%

*Authors’ calculations using SPSS 24.0 software.*

### Procedure and Measures

An electronic survey was conducted during the academic year 2020–2021. Participants were invited by email to complete an online questionnaire (Google forms). The online questionnaire took approximately 20 min to complete and was open after COVID-19-related teaching and learning changes (e.g., transition to digital learning) began.

We apply the mandatory option for responses to all the questions of the survey. Only completed answers are received. The questionnaire was previously tested on 20 students and then improved to collect 200 completed responses as a representative sample according to whole PNU students.

The Princess Nourah Bint Abdulrahman University Institutional Review Board evaluated the proposal considering the national regulations that govern the protection of human’s subjects and has determined that the proposed project poses no more than minimal risk to the participants and approved the project.

The questionnaire contains the following three sections:

Section 1: Digital learning: effectiveness versus traditional learning, costs, capabilities, and educational resources.

Section 2: Digital learning in COVID-19 time, stress, psychological effects, and its role in defense mechanisms and coping strategies.

Section 3: Life satisfaction and attitudes toward COVID-19.

To test the consistency and accuracy of the survey we calculate the Cronbach’s alpha test. Table 2 below presents the Cronbach’s alpha factor for the three sections of the questionnaire, which is around 85%, and indicates that the questions of the survey are computing what they are intended to measure and have a high level of validity and reliability. In addition, the Cronbach’s Alpha factor is calculated for the questions of each section separately to measure the internal consistency of the questions and their reliability to compute what it is designed to measure. Section 1, section 2, and section 3 are perceptively around 85, 81, and 89%. For the rest of the analysis, we apply 1 Sample *t*-Test, Paired Samples Test, and Correlations.

**TABLE 2:** Test of reliability and validity.

Sections	Cronbach’s Alpha coefficient	Number of items
Section-1: Digital learning: effectiveness versus traditional learning, costs, capabilities, and educational resources	0.856	6
Section-2: Digital learning in time of COVID-19, stress, psychological effects and its role in defense mechanisms and coping strategies	0.810	10
Section-3: Life satisfaction and attitudes toward COVID-19	0.891	20
All sections	0.852	36

*Authors’ calculations using SPSS 24.0 software.*

## Results and Discussion

### Digital Learning: Effectiveness versus Traditional Learning, Costs, Capabilities, and Educational Resources

The mean, standard deviation, and one-sample *t*-test are reported for each item in the first section questions (Table 3). The mean displays that the scale of respondents’ thoughts about the enquiry, where 1 strongly disagree to 5 strongly agree. Results testing the hypothesis *H*^1^ confirm that we can reject the null hypothesis, there is a moderate significant difference in the effectiveness, costs, capabilities and educational resources between digital and traditional learning. All the *p*-values are less than 0.05 at confidence level of 95%. Perceptions of students on digital learning have a positive moderate score with means ranging from 2.99 to 3.34 except the first item “*Digital learning is more effective, efficient and productive than traditional learning*” where the mean is equal to 2.31 and shows a low level of agreement. Students who participate in the study confirm that digital learning provides more flexibility in time and allows them to obtain electronic educational resources at a lower cost compared to traditional learning. According to the students, the flexibility and convenience provide by the online course are particularly interesting and attractive features. Digital learning enables student to raise their capabilities by integrating the global digital community which is supported by the university how has the necessary and appropriate technical infrastructure and facilities. This result confirms the finding of Abdulrahim and Mabrouk (2020). Despite all that, the shift from traditional learning to digital learning was declared moderately easy for them. The results of the study testing the first hypothesis illustrate that there is moderate significant difference in effectiveness, costs, capabilities, and educational resources for digital learning compared to traditional learning. In the current literature, similar results were found by Bartley and Golek (2004), Chen et al. (2012), and Agasisti and Johnes (2015), they conclude that cost benefits of digital learning are significantly superior to traditional learning even with social disparities and governments support (Zhang and Worthington, 2017). However, where courses are practical oriented, the fact to shift completely from traditional to digital learning may not be possible (Muthuprasad et al., 2021). Demetriadis and Pombortsis (2007) study the learning efficiency of e-lecture vs. traditional live lecture. Quantitative data of the study show that there are no significant differences in the learning outcomes between students who attended a traditional live lecture and who attended an e-lecture provided they are motivated and the instruction is at an introductory level.

**TABLE 3:** One sample *T*-test (Section 1: Digital learning: effectiveness versus traditional learning, costs, capabilities, and educational resources).

	Mean^1^	Std. deviation	Std. error mean	*t*-value	Sig. (2- tailed)
Digital learning is more effective, efficient and productive than traditional learning	2.315	1.3984	0.0989	23.412	0.000
Digital learning provides more flexibility in time to learn compared to traditional learning	3.215	1.4384	0.1017	31.608	0.000
Digital learning allows students to obtain electronic educational resources at a lower cost compared to traditional learning	3.040	1.4207	0.1005	30.260	0.000
Digital learning enables students to raise their capabilities to adapt to the global digital community	3.345	1.3398	0.0947	35.309	0.000
The university has the necessary and appropriate technical infrastructure and facilities for the application of Digital learning	3.285	1.2496	0.0884	37.176	0.000
The shift from traditional learning to Digital learning was easy for me	2.990	1.4937	0.1056	28.309	0.000

*^1^Analysis of Likert scale (mean score) (mean score: 0.00–1.50, agreement level: Very low; mean score: 1.51–2.50, agreement level: Low; mean score: 2.51–3.50, agreement level: Moderate; mean score: 3.51–4.50, agreement level: High; mean score: 4.51–5.00, agreement level: Very high). Authors’ calculations using SPSS 24.0 software.*

### Digital Learning in Time of COVID-19, Stress, Psychological Effects and Its Role in Defense Mechanisms and Coping Strategies

In the second section of the survey, the questions are elaborated to provide a deeper analysis related to students’ defense mechanisms and coping strategies to face the new educational environment and form of learning. The idea is to search on students’ perceptions and how the COVID-19 and/or the shift to digital learning are affecting their life satisfaction. Table 4 displays the Descriptive Statistics of students’ perceptions of COVID-19 and digital learning. The average mean of the items measuring the student’s perception of digital learning and COVID-19 are 3.59 and 3.53, respectively, and showing a high and almost similar level of agreement.

**TABLE 4:** Descriptive statistics (Section 2: Digital learning in time of COVID-19, stress, psychological effects and its role in defense mechanisms and coping strategies).

		Mean	Std. deviation	Std. error mean
Pair 1	Digital learning causes me anxiety and stress	3.520	1.4422	0.1020
	The COVID-19 pandemic causes me anxiety and stress	3.525	1.2276	0.0868
Pair 2	Exercising helps me reduce the anxiety and stress associated with Digital learning	3.400	1.2360	0.0874
	Exercising helps me reduce the anxiety and stress associated with the COVID-19 pandemic	3.370	1.0812	0.0765
Pair 3	Sleeping enough hours helps me reduce the anxiety and stress associated with Digital learning	3.595	1.3228	0.0935
	Sleeping enough hours helps me reduce the anxiety and stress associated with the COVID-19 pandemic	3.505	1.2877	0.0911
Pair 4	Communicating with friends and classmates helps me reduce the anxiety and stress associated with Digital learning	3.530	1.2437	0.0879
	Communicating with friends, meeting with classmates, helps me reduce the anxiety and stress associated with the COVID-19 pandemic	3.510	1.1819	0.0836
Pair 5	If someone around me succeeds in obtaining a high score, then I am able to obtain a high score	3.930	1.1841	0.0837
	If someone around me gets over the disease (COVID-19 pandemic), I can get over it as well	3.760	1.1219	0.0793

*Authors’ calculations using SPSS 24.0 software.*

All the defense mechanisms are similar and highly perceived (mean around 3.5) to deal with digital learning and the COVID-19. Students affirm that doing sports, sleeping well, and communicating, help them to reduce the anxiety and stress associated with digital learning and COVID-19. This result is supported by the high level of significant positive correlation between pairs 2, 3, and 4, in which, we compare, respectively, how exercising (60%), sleeping (60%), and communicating (63%) face the health crisis and new form of learning at the university (Table 5).

**TABLE 5:** Paired samples test and correlations (Section 2: Digital learning in time of COVID-19, stress, psychological effects and its role in defense mechanisms and coping strategies).

		Mean	Std. deviation	Std. error mean	*T*	Sig. (2-tailed)	Correlation (Sig.)
Pair 1	Digital learning causes me anxiety and stress	−0.0050	1.6488	0.1166	−0.043	0.966	0.245 (0.0000)
	The COVID-19 pandemic causes me anxiety and stress						
Pair 2	Exercising helps me reduce the anxiety and stress associated with Digital learning	0.0300	1.0462	0.0740	0.406	0.686	0.599 (0.0000)
	Exercising helps me reduce the anxiety and stress associated with the COVID-19 pandemic						
Pair 3	Sleeping enough hours helps me reduce the anxiety and stress associated with Digital learning	0.0900	1.1742	0.0830	1.084	0.280	0.596 (0.0000)
	Sleeping enough hours helps me reduce the anxiety and stress associated with the COVID-19 pandemic						
Pair 4	Communicating with friends and classmates helps me reduce the anxiety and stress associated with Digital learning	0.0200	1.0465	0.0740	0.270	0.787	0.629 (0.0000)
	Communicating with friends, meeting with classmates, helps me reduce the anxiety and stress associated with the COVID-19 pandemic						
Pair 5	If someone around me succeeds in obtaining a high score, then I am able to obtain a high score	0.1700	1.2804	0.0905	1.878	0.062	0.384 (0.0000)
	If someone around me gets over the disease (COVID-19 pandemic), I can get over it as well						

*Authors’ calculations using SPSS 24.0 software.*

Results testing the hypothesis *H*^2^ approve that we can accept the null hypothesis, there is no significant difference in how the students deal with the pandemic or with the new form of university learning. All the *p*-values are less than 0.05 at a confidence level of 95%. Finally, the findings show that the coping strategy perception is higher in obtaining a good score and succeeding than to get over the pandemic and recovering from the illness itself. The coefficient of correlation related to pair 5 is around 40%. This means that the students are abler to control their studies better than their health, entirely justified by the nature of the health crisis (Table 5). Findings are similar to Patias et al. (2021), who seek to identify whether there was any relationship between coping strategies adopted by undergraduate Brazilian students in response to social distancing caused by the COVID-19 and symptoms of depression, anxiety, and stress. They find a positive correlation (though small) between some coping strategies and the symptoms of depression, anxiety, and stress. In this regard, being responsible and adopting healthier behaviors can be a way to deal with the situation. The coronavirus pandemic (COVID-19) had undoubtedly psychological impacts on students and will undoubtedly have an impact on their mental health. Lopes and Nihei (2021) conduct an investigation with undergraduate Brazilian students during the COVID-19 pandemic. Their results show that the majority of students surveyed exhibits symptoms of depression, anxiety, stress and lower scores for satisfaction with life. In the same vein, Gori et al. (2021) seek to explore the relationship between anxiety and perceived stress in individuals during confinement related to COVID-19 in Italy. The authors aim to further investigate and study the role of several variables contributing to subjective distress, with a particular focus on the impact of coping strategies and defense mechanisms in catalyzing or hindering the relationship between anxiety and perceived stress in individuals experiencing COVID-19 lock-in.

Students experienced high levels of stress and anxiety during the quarantine period. The COVID-19 epidemic is transforming students’ living and studying conditions and affecting students’ mental health (Rogowska et al., 2020). Results are similar to Chinna et al. (2021) and show that female students in Saudi Arabia experienced significantly higher levels of anxiety compared to their male counterparts.

### Life Satisfaction and Attitudes Toward COVID-19

The third section is related to 2 principal questions, both of them are composed of 10 statements. The first one is on how satisfied you feel about certain aspects of your life, on a scale from 0 to 10. Zero means that you are “not at all satisfied” and 10 means “completely satisfied.” The second is on how you felt yesterday on a scale from 0 to 10. Zero means that you did not experience feelings “at all” yesterday while 10 means that you experienced feelings “all the time” yesterday. This shows 5 items are related to positive feelings and 5 to negative emotions. Results from Tables 6, 7 show that thinking styles affect life satisfaction; there is a significant negative correlation between negative attitudes and life satisfaction of female students during COVID-19 and vice-versa. Results testing the hypothesis *H*^3^ approve that we can accept the alternative hypothesis. There is a significant difference in how attitudes influence the female students’ life satisfaction during COVID-19. All the *p*-values are less than 0.05 at a confidence level of 95%. Results are confirming the literature that a positive attitude influences positively life satisfaction.

**TABLE 6:** Descriptive statistics (Section 3: Life satisfaction and attitudes toward COVID-19).

		Mean	Std. deviation	Std. error mean
Pair 1	Life satisfaction	6.793	1.9571	0.1384
	Positive attitude	6.2013	2.5602	0.1810
Pair 2	Life satisfaction	6.793	1.9571	0.1384
	Negative attitude	6.2141	2.4617	0.1740

*Authors’ calculations using SPSS 24.0 software.*

**TABLE 7:** Paired samples test and correlations (Section 3: Life satisfaction and attitudes toward COVID-19).

	Mean	Std. deviation	Std. error mean	*t*	Sig. (2-tailed)	Correlation (Sig.)	
Pair 1	Life satisfaction and positive attitude	0.5917	1.7643	0.1247	4.743	0.000	0.726 (0.0000)
Pair 2	Life satisfaction and negative attitude	0.5788	3.6151	0.2556	2.264	0.025	−0.330 (0.0000)

*Authors’ calculations using SPSS 24.0 software.*

As a matter of fact, Duong (2021), found that COVID-19 fear and anxiety reduced life satisfaction and increased sleep disturbance via psychological distress. Indeed, COVID-19 fear and anxiety were strongly related to psychological distress, sleep disturbance, and life satisfaction. The psychological impact of the COVID-19 pandemic on university life is more extensive and intense than psychosomatic, social, and economic dimensions (Karakose et al., 2021).

In an epidemic context, family support can play a major role in the attitude toward COVID-19 particularly concerning social distancing and avoidance of social interactions. Family support is also important for maintaining good mental health and could play an essential role in helping people cope with the stress and difficulties caused by COVID-19. Li and Xu (2020) examine the role of family support during the COVID-19 pandemic among Chinese people. They find that closer ties with family can provide support for people and help maintain positive mental health. Cao et al. (2020) show that living with parents is a factor that can reduce anxiety. Moreover, they find that social support is negatively associated with anxiety symptoms of college students during the COVID-19 pandemic. Krause et al. (2021) investigate whether media use was related to changes in life satisfaction of Australian university students at the beginning of the COVID-19 pandemic. They explore different types of media use and they highlight the potential benefits of music listening on life satisfaction during COVID-19. Similarly, the study of Gori et al. (2021) confirms the significant impact of state anxiety levels on perceived stress and shows that positive thinking, spirituality, and conviction can have a strong positive influence on the well-being of individuals.

## Conclusion, Suggestions, and Limitations

In this study, we examined the relationship between digital learning, life satisfaction, and perceived stress due to the COVID-19 emergency among 200 students in Saudi Arabia. We found that digital learning provides more flexibility in time and allows students to obtain electronic educational resources at a lower cost compared to traditional learning. In addition to this, coping strategy perception is higher in obtaining a good score and succeeding than to get over the pandemic and recovering from the illness itself. Finally, a positive attitude stimulus positively life satisfaction.

The results of this study provide some practical implications. While all universities have opted to continue teaching, the pandemic has also revealed the central role that education plays in our universities. There is an urgent need for universities to revalue people involved in it. Universities must immediately take into consideration mental health and workload for students and member faculties by implementing mental health action plans and adapting working conditions. There is a need to reduce workload and invest in teaching and learning, coaching, and psychological support. The COVID-19 crisis has shown the resilience and facilities that universities can offer. However, it also showed the inequalities that not only exist but have been exacerbated by the pandemic. It is crucial to move forward and rethink how to make the higher education system better able to fulfill its mission in the future.

However, this study has some limitations, we focus only on female students, and we suppose that education with digital learning methods is carried out only because of a pandemic and does not at all discuss digital learning as a communication method due to the development of information technology. Future research will focus on digital learning and information technology development in higher education system such as both sexes.

## Data Availability Statement

The raw data supporting the conclusions of this article will be made available by the authors, without undue reservation.

## Author Contributions

FM implemented the survey and collected data. FM, MM, and AA contributed to writing—original draft and writing—reviewing and editing the sections of the manuscript. FM performed the formal analysis. MM managed and coordinated the research activity planning and execution. All authors collaborated to approve the final version of the manuscript.

## Conflict of Interest

The authors declare that the research was conducted in the absence of any commercial or financial relationships that could be construed as a potential conflict of interest.

## Publisher’s Note

All claims expressed in this article are solely those of the authors and do not necessarily represent those of their affiliated organizations, or those of the publisher, the editors and the reviewers. Any product that may be evaluated in this article, or claim that may be made by its manufacturer, is not guaranteed or endorsed by the publisher.
